# P46 Abdominal pain in newly diagnosed lupus

**DOI:** 10.1093/rap/rkae117.077

**Published:** 2024-11-05

**Authors:** Ann Ninan, Gwenan Huws

**Affiliations:** Royal Gwent Hospital, Newport, United Kingdom; Royal Gwent Hospital, Newport, United Kingdom

## Abstract

**Introduction:**

This case describes an unusual presentation of lupus in a young female patient who had presented several times to hospital with arthralgia and fevers. She developed acute abdominal pain and vomiting with a raised amylase and was diagnosed with lupus pancreatitis; then subsequently developed acute respiratory failure, requiring invasive ventilation and ECMO. This case was significant in highlighting a rare symptom of lupus, which should be considered in patients with non-specific abdominal pain, and the complications that can arise such as ARDS and diffuse alveolar haemorrhage.

**Case description:**

A 21 year old female student of Asian descent, who was fit and well with no previous medical history. She described a 1 year history of mild Raynaud’s and intermittent wrist pain. She had two successive hospital admissions after developing fevers, significant arthralgia/myalgia, weight loss and vomiting after a recent viral URTI. She was found to have a raised ANA 1:1280, ds-DNA 71.1, positive anti-Ro, Sm and RNP antibodies. She was discharged with urgent rheumatology follow up and outpatient investigations for vomiting.

She was admitted for a third time shortly afterwards with ongoing abdominal pain and vomiting; a CT showed acute pancreatitis, with serum amylase was raised at 629 U/L and a Glasgow-Imrie score of 3- high risk for severe pancreatitis. Despite 7 days of IV antibiotics and fluids, she was no better, and was then reviewed by rheumatology as an inpatient. She had evidence of active lupus with malar rash, joint pains, fevers, rising ds-DNA titres, low C3/C4, giving a SLEDAI of 17. A CT thorax showed pleural effusions and bilateral ground glass changes in both lungs.

Overall, an infective cause was felt to be less likely, and therefore she underwent treatment with IV methylprednisolone, and hydroxychloroquine, and a few days later had 5 days of intravenous immunoglobulins.

Unfortunately she deteriorated with ongoing vomiting and new respiratory failure with haemoptysis. She had invasive ventilation for a suspected diagnosis of either diffuse alveolar haemorrhage, lupus pneumonitis or ARDS secondary to pancreatitis. She was transferred to a London hospital for ECMO due to further respiratory deterioration, and there she subsequently had cyclophosphamide and rituximab, with good clinical improvement. She has since been discharged after a prolonged admission, and has resumed her studies, attends the gym regularly and is now on a maintenance treatment of prednisolone, MMF and hydroxychloroquine.

**Discussion:**

This was an interesting case showing a clear multisystem disease process which progressed fairly rapidly; unfortunately she did not have a face to face review by rheumatology until the third presentation to hospital, leading to a delay in diagnosis and treatment.

Pancreatitis is a rare but well documented symptom in lupus, and should be considered as a diagnosis in lupus patients who present with abdominal pain. The symptoms may be atypical with generalized abdominal pain that may not have the classical description of central abdominal pain which radiates through to the back. Subacute presentations may show raised pancreatic enzymes without typical clinical symptoms.

Treatment is counter-intuitive to normal cases of pancreatitis, where glucocorticoids are a known risk factor for development of pancreatitis; however in lupus populations, steroids should be started if the diagnosis is suspected, and is shown to have an associated lower mortality than patients not prescribed steroids. Plasmapharesis and IVIG may be helpful in severe cases of pancreatitis.

These are also both options in managing diffuse alveolar haemorrhage (DAH). This condition is a severe respiratory complication of SLE, and is a rapidly progressive condition with an estimated mortality of 50%. DAH should be suspected in patients with dyspnoea, haemoptysis and worsening pulmonary infiltrates. Pulmonary function testing can confirm raised gas transfer values due to alveolar haemorrhage.

**Key learning points:**

• One of the key learning points in this case was investigating the symptoms of persistent nausea and vomiting in a patient with newly diagnosed lupus. Lupus pancreatitis is estimated to affect 0.7-4% of patients and is more likely to appear as an initial or early symptom of lupus. Given the variability in symptoms with lupus-associated pancreatitis, this diagnosis should be considered in patients who have acute pancreatitis in the absence of typical risk factors (gallstones, alcohol, steroid use). Particularly for younger female patients with idiopathic pancreatitis, early testing for lupus may identify a diagnosis before other typical clinical symptoms of lupus are evident.

• Other forms of autoimmune pancreatitis can be due to conditions such as IgG4 disease, and have a distinct phenotype which mimics pancreatic carcinoma rather than acute pancreatitis. However this highlights the importance of considering GI involvement during work up for patients with suspected multisystem disorders.

• There are no guidelines for the dosage and duration of steroid therapy in lupus-associated pancreatitis. Other immunosuppressive agents such as azathioprine and cyclophosphamide have been used, with IVIG and plasmapheresis suggested in high severity cases.

I would welcome discussion on others’ experience in treating this rare symptom of lupus, and as a specific learning point, any guidance on steroid therapy.

